# A Two Phase Method for Solving the Distribution Problem in a Fuzzy Setting

**DOI:** 10.3390/e21121214

**Published:** 2019-12-11

**Authors:** Krzysztof Kaczmarek, Ludmila Dymova, Pavel Sevastjanov

**Affiliations:** Department of Computer Science, Czestochowa University of Technology, Dabrowskiego 73, 42-201 Czestochowa, Poland; krzysztof.kaczmarek@icis.pcz.pl (K.K.); dymowa@icis.pcz.pl (L.D.)

**Keywords:** fuzzy extension, simplex method, fuzzy multiobjective distribution problem, aggregation of aggregating modes

## Abstract

In this paper, a new method for the solution of distribution problem in a fuzzy setting is presented. It consists of two phases. In the first of them, the problem is formulated as the classical, fully fuzzy transportation problem. A new, straightforward numerical method for solving this problem is proposed. This method is implemented using the α-cut approximation of fuzzy values and the probability approach to interval comparison. The method allows us to provide the straightforward fuzzy extension of a simplex method. It is important that the results are fuzzy values. To validate our approach, these results were compared with those obtained using the competing method and those we got using the Monte–Carlo method. In the second phase, the results obtained in the first one (the fuzzy profit) are used as the natural constraints on the parameters of multiobjective task. In our approach to the solution of distribution problem, the fuzzy local criteria based on the overall profit and contracts breaching risks are used. The particular local criteria are aggregated with the use of most popular aggregation modes. To obtain a compromise solution, the compromise general criterion is introduced, which is the aggregation of aggregating modes with the use of level-2 fuzzy sets. As the result, a new two phase method for solving the fuzzy, nonlinear, multiobjective distribution problem aggregating the fuzzy local criteria based on the overall profit and contracts breaching risks has been developed. Based on the comparison of the results obtained using our method with those obtained by competing one, and on the results of the sensitivity analysis, we can conclude that the method may be successfully used in applications. Numerical examples illustrate the proposed method.

## 1. Introduction

The problem of optimal distribution and the transportation problem are similar mathematical formulations and may be considered as the particular cases of the general linear programming problem. The first effective algorithm for solution of transportation problem was developed in 1979 by Isermann [1]. Since then a lot of papers devoted to this problem in the case of real-valued parameters were published in the scientific journals.

Nevertheless, in practice, we often meet different kinds of uncertainty when the parameters of these optimization problems are presented by intervals or fuzzy values. In [2], Zimmermann first presented the mathematical formulation of fuzzy linear programming problem (*FLPP*) with its approximate solution.

Based on the literature analysis, we can say that for the solution of *FLPP*, two main approaches seem to be dominating.

In compliance with the first of them, a fuzzy linear programming problem is solved with the suggestion that decision variables are real values. Therefore, the real-valued solution of fuzzy problem is obtained. The real-valued solutions of *FLPP* were obtained with different simplifications of the form of fuzzy parameters in [3,4,5]. In [6,7], the approximate solutions of fuzzy transportation problem were obtained with the assumption that decision variables are real-values.

In the framework of second approach, the decision variables are assumed to be fuzzy values. Therefore, a fuzzy solution of FLPP is obtained. The *FLPP* problem with all fuzzy parameters and decision variables is named a fully fuzzy linear programming (*FLPP*) problem. It is clear that such an approach is more natural because it provides more useful information for decision makers, since a real-values solution is less informative than a fuzzy one. On the other hand, this approach needs a more general problem formulation and the solutions of more difficult, additional mathematical problems.

In the relatively few papers devoted to *FLPP* problems, only approximate solutions have been presented. Usually some simplifications of fuzzy parameters are used (triangular or trapezoidal fuzzy numbers, L−R fuzzy numbers, and so on) [8,9,10].

Of course, it is hard to estimate all possible negative consequences of above-mentioned restrictions and simplifications. Therefore, the approaches based on the α-cuts representation of fuzzy values seem to be more promising because they are free of any restrictions on the shape of fuzzy values used for the solution of real-world problems.

In [11], the solution of fully fuzzy reverse logistics network design (*FFRLND*) problem is presented. A new parametric method for the solution of the problem in the form of fuzzy decision variables is proposed. The method is based on the α-cuts representation of fuzzy numbers. Unfortunately, the ranking function used for the fuzzy values comparison (*FVC*) based on the comparing of the mean values of triangular fuzzy numbers seems to be too simplified, whereas *FVC* plays an important role in the solution of the problem considered. In [12], the detailed literature review on fuzzy transportation problem is presented. Taking into account the restrictions and drawbacks of known methods, the authors proposed a direct approach to the solution of fully fuzzy transportation problems. The method is based on the α-cuts representation of fuzzy values and fuzzy decoding procedure based on constrained fuzzy arithmetic operations and a fuzzy ranking technique. In our opinion, the use of constrained fuzzy arithmetic and simplified fuzzy ranking technique makes it possible to avoid some known problems, but generates new ones.

Based on the above literature analysis and especially on the comprehensive literature review presented recently in [12], we can say that the most informative solutions of fuzzy linear transportation problem and fuzzy linear distribution problem having similar mathematical formulations that may be obtained in the framework of fully fuzzy linear transportation problem *FFLTP*. In this framework, all or part of parameters are fuzzy values and the decision variables are fuzzy values too. There are many approximate solutions of fully fuzzy linear programming problem based on different simplifications of fuzzy parameters proposed in the literature. It is hard to estimate the possible negative consequences of such simplifications when we deal with real-world problems. Therefore, it seems to be promising to use approaches based on the α-cut representation of fuzzy numbers with corresponding operations on them and an adequate method for fuzzy numbers’ comparison. Nevertheless, we found in the literature, only two papers [11,12] in which this approach was used. Unfortunately, in these papers, the so-called constrained fuzzy arithmetic, which has many undesirable properties, was applied. The extremely simplified method for the fuzzy numbers’ comparison based only on the means of triangular fuzzy numbers was used. So we can say that the methods proposed in these papers should be assumed to be rather unreliable ones.

It is important to note that in all considered cases the fuzzy distribution problem was treated as a fuzzy, single-criterion task with fuzzy constraints. On the other hand, oppositely to the classical transportation problem, the fuzzy constraints in the distribution problem may be naturally treated as local fuzzy criteria of contracts breaching risks, and therefore, a more specified and correct form of the fuzzy distribution problem is a non-linear fuzzy multiobjective task. For its formulation, the corresponding criterion of profit maximization is needed. Such a criterion may be obtained on the base of solution of the fully fuzzy distribution problem in the form of fuzzy single-criterion task with fuzzy constraints, which provides the optimal fuzzy profit. It is worth noting that we did not find similar approaches in the literature. Therefore, we have two phases of the solution to our problem.

In the fist of them, we obtain the solution of fully fuzzy single-criterion linear distribution problem (*FFDP)* with fuzzy constraints. In the second phase, using the results obtained in the first one, the non-linear fuzzy multiobjective distribution problem *FDP* is formulated and solved.

In the first phase, to avoid the above-mentioned shortcomings of known approaches, a new approach to the solution of fully fuzzy linear distribution problem (*FFDP*) based on the straightforward fuzzy extension of the simplex method is proposed. The traditional fuzzy arithmetic rules with probabilistic approach to the interval and fuzzy values comparison are applied in this fuzzy extension. The use of α-cut representation of fuzzy values allows us to avoid any simplifications of their forms. The proposed fuzzy extension is implemented using an object-oriented technique.

In the second phase, the multiobjective non-linear fuzzy distribution problem is formulated. The local criterion of profit maximization is introduced using a fuzzy optimal profit obtained at the first phase as a range of achievable profits. To obtain a general criterion, all local criteria are aggregated, taking into account their relative importance. The local criteria are aggregated with the use of most popular aggregation modes. To obtain a compromise solution, the general compromise criterion is introduced, which is the aggregation of aggregating modes with the use of level-2 fuzzy sets. Finally the solution is obtained using a numerical method.

The main advantages of the proposed method in comparison with the existing ones are as follows:

- The straightforward fuzzy extension of simplex method allows us to avoid the use of rather unreliable meta-heuristic method used for the solution of *FFLTP* in the competing approach proposed in [12].
- The treatment of fuzzy constraints as local risk criteria reflects better the specificity of *FDP*, and provide the opportunity to formulate the *FDP* for the first time as the fuzzy nonlinear multiobjective problem aggregating the fuzzy local criteria based on the overall profit and contracts breaching risks.
- Since there are many possible methods for aggregating the fuzzy local criteria proposed in the literature, the problem of finding a compromise solution arises, which in the framework of this approach, for the first time, is solved on the basis of aggregation of aggregating modes with the use of level-2 fuzzy sets.

The reminder of paper is set out as follows.

Section 2 is devoted to the first phase: straightforward fuzzy extension of the single-criterion distribution problem. The mathematical tools used for the fuzzy extension of simplex method are presented. The selection of the method for fuzzy values comparison is carefully argued since it plays a pivotal role in the implementation of the fuzzy simplex method. The *FFDP* is formulated and solved. It is shown how the initial *FDP* is transformed to the canonical form of *FLPP*. The results obtained with the use of the developed method and their comparison with those obtained using competing method and Monte–Carlo method are presented using illustrative examples. The sensitivity analysis of model is provided as well. In Section 3, the results obtained at the firs phase are used to define the local criteria needed to formulate the multiobjective fuzzy distribution problem. To formulate this problem, the different aggregating modes and their aggregation on the basis of the level-2 fuzzy sets are used to aggregate initial local criteria. The solution is obtained using the direct random search method. The illustrative examples are presented. Conclusions are covered in Section 4.

## 2. The First Phase: Straightforward Fuzzy Extension of *FDP*

At this phase of the solution of *FDP*, the problem is formulated as a linear fully fuzzy single-criterion distribution problem. Since in this case, the problem has a mathematical structure similar to the structure of fully fuzzy transportation problem, for its solution a straightforward fuzzy extension of simplex method is used. The mathematical structure of the usual simplex method is extended by replacing all parameters with fuzzy ones and all arithmetic operations with corresponding operations on fuzzy values including operations of interval and fuzzy values comparisons. It is clear that in such an approach to the fuzzy extension of the simplex method, the correctness of the fuzzy arithmetic operations and very important fuzzy values’ comparisons play key roles in obtaining correct results of the optimization.

### 2.1. Mathematical Tools

We can say that fuzzy arithmetic is a well studied and formalized part of fuzzy sets theory. On the other hand, when we deal with the implementation of fuzzy arithmetic rules, we meet some problems. In the most general form, the fuzzy rules are based on the so-called extension principle proposed by Zadeh [13]. An arbitrary *t*-norm is used in the mathematical formalization of this principle. Suppose *X*, *Y*, and *Z* are fuzzy values with the corresponding membership functions μ(x), μ(y), μ(z) (x∈X, y∈Y, and z∈Z) and @∈{+,−,∗,/} being an arithmetical operation. Then,

(1) $$Z=X@Y=\{z=x@y,\mu \left(z\right)=\max_{z}t\left(\mu \left(x\right),\mu \left(y\right)\right),x\in X,y\in Y\}.$$

According to the studies of Zimmermann and Zysno [14], the choice of an appropriate *t*-norm is rather a context dependent problem.

Another, more practical approach to the mathematical representation of fuzzy arithmetic rules is based on the α-cut presentation of fuzzy numbers [15].

If *X* is a fuzzy value, then $X=\underset{\alpha }{⋃}\alpha X_{\alpha }$, where $\alpha X_{\alpha }$ is the fuzzy subset: x∈U, $\mu_{X}\left(x\right)\geq \alpha$, $X_{\alpha }$ is the support set of fuzzy subset $\alpha X_{\alpha }$ and *U* is the universe of discourse. It is very important that for fuzzy values *X* and *Y*, all the mathematical operations on them may be represented as sets of operations on intervals presenting their α-cuts:

(2) $${\left(X@Y\right)}_{\alpha }=X_{\alpha }@Y_{\alpha }.$$

At fist glance, the straight α-cut representation of fuzzy arithmetic rules values seems to be a rough one when comparing with the general expression (1). But in the practical numerical implementation of (1) the discretization of the supports of considered fuzzy values *X*, *Y*, and *Z*, especially when we have complicated forms of μ(x) and μ(y), is inevitable. It is shown in [16], that any discretization of (1) provides unacceptable non-convex results. It seems natural that the results obtained with the use of the general Definition (1) may be somewhat improved using the more detailed discretization, but in practice a very dense disretization is usually needed to do that. It is worth noting here that undesirable results were obtained with the use of different *t*-norm and arithmetical operations, whereas when using the α-cut presentation of fuzzy arithmetic rules we have no such problems at all. For more analysis and details, see for an example, [16]. Therefore, here it is quite enough for our purposes to state that there are some difficult practical problems when using general expression (1) and that there are none these problems in the case of an α-cut representation of fuzzy arithmetic rules. So we can say that the α-cut representation of fuzzy arithmetic rules may be recognized as a reliable basis for mathematical modeling in the fuzzy setting.

It is seen that fuzzy arithmetic rules presented by α-cuts are based on interval arithmetic rules. Therefore, the presentation of basic definitions of applied interval analysis should be provided.

There are may internal methodological problems in the framework of interval analysis. Therefore, several different approaches to the formulation of interval arithmetic rules were proposed in the literature [17,18]. All of them have own drawbacks and restrictions. Therefore, here we will use the classical definition of interval arithmetic which seems to be more logically justified and usually used in the solution of real-world problems. This basic definition may be presented as follows. Let $X=[x_{1},x_{2}]$ and $Y=[y_{1},y_{2}]$ be intervals, @∈{+,−,∗,/}. Then, the resulting interval *Z* is calculated using the following expression:

(3) Z=X@Y={z=x@y,x∈X,y∈Y}.

It is clear that this definition provide the results based only on the bounds of intervals:

(4) $$\begin{array}{c} X+Y=[x_{1}+y_{1},x_{2}+y_{2}],\\ \Sigma X-Y=[x_{1}-y_{2},x_{2}-y_{1}],\\ XY=[min\left(x_{1}y_{1},x_{2}y_{2},x_{1}y_{2},x_{2}y_{1}\right),max\left(x_{1}y_{1},x_{2}y_{2},x_{1}y_{2},x_{2}y_{1}\right)],\\ X/Y=\left[x_{1},x_{2}\right]\left[1/y_{2},1/y_{1}\right],0\notin Y. \end{array}$$

As important role in the solution of fully fuzzy transportation and distribution problems plays the operation of fuzzy values comparison. Since our approach is based on the α-cut presentation of fuzzy numbers, an appropriate method for interval comparison should be chosen.

Most methods for interval comparison are based on the real-valued representation of intervals Ref. [19,20,21]. Moreover, Wang et al. [22] showed that usually, these methods are based on the comparison on interval means. Of course, such simplifications of the problem may provide in practice unpredictable undesirable results. Therefore, in [22] the authors proposed a heuristic method which is not based on the above-mentioned simplifications. Let $Y=[y_{1},y_{2}]$ and $X=[x_{1},x_{2}]$, be crisp intervals; then, the possibility that Y≥X may be, according to [22], calculated as follows:

(5) $$Po\left(Y\geq X\right)=\frac{\max\left\{0,y_{2}-y_{1}\right\}-\max\left\{0,y_{1}-x_{2}\right\}}{x_{2}-x_{1}+y_{2}-y_{1}}.$$

Unfortunately, this approach does not provide the separated interval equality relation, because for X=Y, i.e., $x_{1}=y_{1},x_{2}=y_{2}$ from (5), we get Po(Y≥X)=0.5,P(Y≤X)=0.5. In the literature, interval equality relation is sometimes treated as identity [18], or only in conjunction with interval inequality [23] or even as an impossible relation [24]. On the other hand, in accordance with the classical definition [18], an interval *X* is entirely represented by its bounds ($X=[x_{1},x_{2}]$). Therefore, the interval *X* may be considered as a mathematical object completely defined by the pair $x_{1},x_{2}$. Then it is clear that if we have two such objects *X* and *Y* defined by equal bounds ($x_{1}=y_{1},x_{2}=y_{2}$) we can declare that *X* and *Y* are equal intervals.

Let us consider some hypothetical measure me(X@Y)∈[0.1] (@∈{>,=,<}) of interval equality/inequality. The logically justified properties of me(X@Y) may be only such that me(X=Y)=1, me(X>Y)=0, and me(X<Y)=0 for equal intervals *X* and *Y*, and me(X=Y)=0 for the completely different *X* and *Y* when they have no intersection.

Therefore, to develop a measure of interval equality/inequality with defined above desirable properties, we employ the so-called probabilistic approach to interval comparison. This idea is not a new one. The review of methods based on the probabilistic approach to the interval comparison is made in [19]. However, only in [25] was the consistent set of interval and fuzzy values relations comprising separated equality and inequality relations first developed with the use of the probability approach. The attractiveness of this approach is the possibility to obtain a complete set of probabilities Pr(X>Y),Pr(X<Y), and Pr(X=Y) for the intervals *X* and *Y* when compared using the only one postulation—that the supports of *X* and *Y* are uniform distributions of random values x∈X,y∈Y. It is worth emphasizing here that only in [19,25] do the developed expressions provide in any case Pr(X>Y)+Pr(X=Y)+Pr(X<Y)=1. In [19], two possible propositions related to conditional probabilities were used to get two sets of interval relations named as “weak” and “strong” relations. We will use here, only the “strong” relations (the probabilities of X>Y, X<Y, and X=Y) since they are an asymptotic case of the general approach based on the Dempster–Shafer theory (see [19]). These expressions are presented as follows.

**Figure 1:**
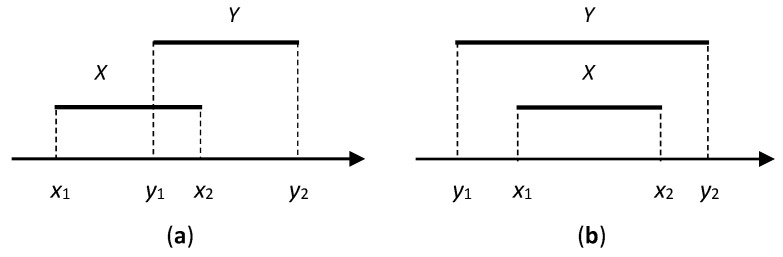
Non trivial interval relations. (**a**) Overlapping case and (**b**) inclusion case.

In the case of overlapping intervals (see Figure 1a):

(6) $$Pr\left(Y<X\right)=0,Pr\left(Y=X\right)=\frac{{\left(x_{2}-y_{1}\right)}^{2}}{\left(x_{2}-x_{1}\right)\left(y_{2}-y_{1}\right)},Pr\left(Y>X\right)=1-Pr\left(Y=X\right).$$

In the case of including the interval (see Figure 1b):

(7) $$Pr\left(Y<X\right)=\frac{x_{1}-y_{1}}{y_{2}-y_{1}},Pr\left(Y=X\right)=\frac{x_{2}-x_{1}}{y_{2}-y_{1}},Pr\left(Y>X\right)=\frac{y_{2}-y_{1}}{y_{2}-y_{1}}.$$

It is easy to see that in the both cases, Pr(X<Y)+Pr(X=Y)+Pr(X>Y)=1.

The expressions for the fuzzy values comparison were obtained in [19] with the use of interval relations (6) and (7), and the α-cut representation of fuzzy values. Suppose $\hat{X}$ and $\hat{Y}$ are the fuzzy numbers on *Z* with the corresponding membership functions $\mu_{X}\left(z\right)$, $\mu_{Y}\left(z\right)$: Z→[0,1]. Then, $\hat{X}$ and $\hat{Y}$ may be represented by the sets of α-cuts $\hat{X}$ = $\underset{\alpha }{⋃}X_{\alpha }$, $\hat{Y}$ = $\underset{\alpha }{⋃}Y_{\alpha }$, where $X_{\alpha }$ = $\left\{z\in Z:\mu_{X}\left(z\right)\geq \alpha \right\}$, $Y_{\alpha }$ = $\left\{z\in Z:\mu_{Y}\left(z\right)\geq \alpha \right\}$ are the crisp intervals. With the use of α-cuts representation, the fuzzy relations $\hat{X}$rel$\hat{Y}$, rel∈{<,=,>} may be presented as follows:

(8) $$\hat{X}\;rel\;\hat{Y}=\underset{\alpha }{⋃}X_{\alpha }\;rel\;Y_{\alpha }.$$

Because $X_{\alpha }$ and $Y_{\alpha }$ are crisp intervals, the probability $Pr_{\alpha }\left(X_{\alpha }>Y_{\alpha }\right)$ for all pairs $X_{\alpha }$ and $Y_{\alpha }$ should be obtained from (6) and (7). Obviously, the set of the probabilities $Pr_{\alpha }$, (α∈(0,1]) may be considered as the support of fuzzy subset

(9) $$Pr\left(\hat{X}>\hat{Y}\right)=\left\{\frac{\alpha }{Pr_{\alpha }\left(X_{\alpha }>Y_{\alpha }\right)}\right\},$$

where the value of α is treated as the degree of membership to the fuzzy value $P(\hat{X}>\hat{Y})$. Similarly, the fuzzy subset $Pr(\hat{X}=\hat{Y})$ may also be obtained. It is easy to show that in overlapping case $Pr(\hat{X}>\hat{Y})$ + $Pr(\hat{X}=\hat{Y})$ = “near 1”, and in inclusion case $Pr(\hat{X}>\hat{Y})$ + $Pr(\hat{X}=\hat{Y}$+ $Pr(\hat{X}<\hat{Y})$ = “near 1” (“near 1” is a symmetrical fuzzy value centered around 1. In applications, often the real-valued representations of fuzzy numbers are used to compare fuzzy values. Therefore, different characteristic numbers of fuzzy set may be used. Nevertheless, it seems enough justified to apply the defuzzification, which for the set of α-cuts used, may be presented as follows:

(10) $$\bar{P}r\left(\hat{X}>\hat{Y}\right)=\frac{\sum_{\alpha }\alpha \cdot Pr_{\alpha }\left(X_{\alpha }>Y_{\alpha }\right)}{\sum_{\alpha }\alpha }.$$

That last expression is based on the natural assumption that the α-cut contribution to the final probability assessment is increasing with the rise of α.

### 2.2. The Fully Fuzzy Extension of Simplex Method

**Figure 2:**
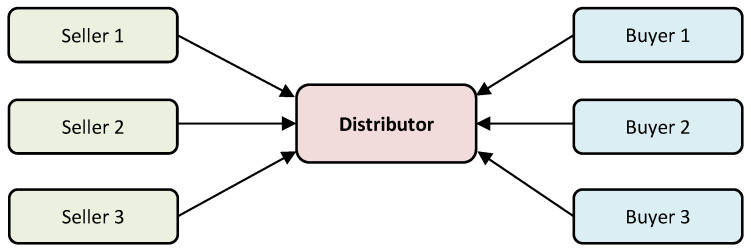
The distributing transactions.

Oppositely to the transportation task, in the distribution problem we try to maximize the final profit of distributor taking into account transportation costs, prices of sellers and buyers, and restrictions concerned with contracts which may be signed by the distributor with sellers and buyers. Let us assume that the distributor has *M* possible sellers and *N* possible buyers (Figure 2).

Let $a_{i}$, *i* = 1 to *M*, be the maximal quantities of products which may be sold by sellers, and $b_{j}$, *j* = 1 to *N*, be the maximal quantities of products that may be bought by the buyers. The fuzzy profit $\hat{p}r_{ij}$ is the result of supplying of a product unit from *i*th seller to *j*th buyer and can be presented as follows: $\hat{p}r_{ij}=c_{j}-c_{i}-{\hat{t}}_{ij}$, where $c_{j}$ and $c_{i}$ are the prices of selling and buying, respectively; ${\hat{t}}_{ij}$ is the fuzzy cost of supplying of a products unit from *i*th seller to *j*th buyer. On the basis of the signed contracts, the distributor must buy at least $p_{i}$ products units at the price of $c_{i}$ monetary units for unit of product from each *i*th seller and to sell at least $q_{j}$ product units at price of $c_{j}$ monetary units for unit of product to each *j*th buyer. Such constraints $p_{i}$ and $q_{j}$ restrict only the lower bounds of permissible optimal quantities of product to be bought and sold. Hence, these quantities can be negotiated, and hereinafter, we will consider them as the fuzzy constraints ${\hat{p}}_{i}$, ${\hat{q}}_{j}$. So, the problem is the optimization of product quantities ${\hat{x}}_{ij}$ (*i* = 1, …, *M*; *j* = 1, …, *N*) supplying from *i*th seller to *j*th buyer consumer which maximize the summarized fuzzy profit of distributor $\hat{P}r$ taking into account the fuzzy constraints:

(11) $$\hat{P}r=\sum_{i=1}^{M}\sum_{j=1}^{N}\left(\hat{p}r_{ij}{\hat{x}}_{ij}\right)\to \max,$$

(12) $$\sum_{j=1}^{N}{\hat{x}}_{ij}\leq a_{i}\;\left(i=1..M\right),\sum_{i=1}^{M}{\hat{x}}_{ij}\leq b_{j}\;\left(j=1..N\right),$$

(13) $$\sum_{j=1}^{N}{\hat{x}}_{ij}\geq {\hat{p}}_{i}\;\left(i=1..M\right),\sum_{i=1}^{M}{\hat{x}}_{ij}\geq {\hat{q}}_{j}\;\left(j=1..N\right).$$

In this model, the parameters $a_{i}$ and $b_{j}$ are represented by real values since they represent the maximal possible quantities of product which a seller agrees to sell and the maximal quantities of product which a buyer is ready to buy. It is clear that in practice, the values of parameters $a_{i}$ and $b_{j}$ are usually known as strong starting limits and are not negotiated.

It is seen that the above model from mathematical point of view is the same as the fully fuzzy transportation problem. Hence, for its solution, the fuzzy extension of the simplex method can be applied. Here, we only briefly describe the most important steps of implementation of this method.

First, of all the parameters of the model are substituted with the corresponding fuzzy values and all arithmetical rules, including operation of comparison, are substituted with the fuzzy operations defined in the previous subsection using the α-cut representation of fuzzy values. To transform the model (11)–(13) into its canonical form, we substitute the two-index representation of this model for the single-index one. Replacing the inequalities by equalities we transform the model into its augmented form.

The next step is the presentation of the augmented form in the canonical form. Introducing the so-called slack variables, these inequalities are transformed to the set of equalities in the canonical form. What we avoid here is the presentation of routine procedure of transformation of the initial fuzzy linear programming problem to the canonical form using corresponding mathematical expressions. These expressions are far too cumbersome to be appropriate for the scientific paper. Moreover, such a procedure (in its non-fuzzy form) is thoroughly described in textbooks.

The next steps of the proposed approach are formally the same as in the standard simplex method, but are implemented in the fuzzy form. For the implementation of developed approach, the technique of object-oriented programming was used. For this purpose, the special class “Fuzzy value” was implemented on the base of language C++. This class comprises the overloaded operators which represent the operations on fuzzy values. In this approach, the fuzzy parameters and variables are implemented as the objects of class “Fuzzy value”. We can say that the algebraic structure of fuzzy extended simplex method is formally the same as the algorithm of the usual simplex method.

To validate our approach, we compared it with some other methods for the solution of fully fuzzy transportation or distribution problems. We found in the literature, only one paper [12] where such a problem was solved using an α-cut representation of fuzzy values, the constrained interval arithmetic operations, and a fuzzy ranking technique based on the real-valued means of fuzzy values. Since the constrained interval arithmetic is in contradiction with the logically justified basic definition (3) which is successfully used in the solution of real-world problems, and taking into account the extremely simplified ranking technique which leads to the loss of important information, we can say that the method proposed in [12] may provide undesirable results.

Therefore, when comparing the method proposed in [12] with our approach, we should obtain different results, since substantially different mathematical tools were used. To estimate this possible difference and validate our approach, consider the example from [12]. The fully fuzzy transportation problem with triangular fuzzy numbers was solved in the case of three sources and four destinations. It was formulated as follows:

Min$\hat{Z}=\sum_{i=1}^{3}\sum_{j=1}^{4}\hat{c_{ij}}\hat{x_{ij}}$ s.t.: $\sum_{i=1}^{3}{\hat{x}}_{ij}={\hat{a}}_{j},j=1,2,3,4;$$\sum_{j=1}^{4}{\hat{x}}_{ij}={\hat{b}}_{i},i=1,2,3$.

The fuzzy parameters of above problem are presented in Table 1.

In [12], the efficient solutions of the problem being consideredd are presented only on the particular α-cuts (α = 0, α = 0.5, α = 1). Therefore, in Table 2 they are presented in the crisp interval form. The corresponding results that we have obtained using our approach are shown in Table 3.

Comparing the results presented in Table 2 and Table 3, we can conclude that they coincide on the qualitative level, but our results seem to be a bit more variable. Therefore, what we can say is that our results are likely more reliable, as they were obtained using more justified mathematical tools.

On the other hand, our problem (11)–(13) may be solved using Monte–Carlo method. Of course this method is extremely expensive, and therefore can be used only for a few number of sellers and buyers. It is important that for its implementation, interval and fuzzy arithmetic operations are not needed. Obviously, the results obtained using this method may serve as in some sense as an empirical basis for the other methods’ validations.

Therefore, to validate our method, the results of *FDP* solution based on the straightforward fuzzy extension of simplex method were compared with those obtained using Monte–Carlo for the problem (11)–(13). In the framework of Monte–Carlo approach, all uncertain parameters are treated as random values with normal distributions. The usual Monte–Carlo procedure was applied; i.e., for each complete set of randomly chosen values according to corresponding normal frequency distributed, real-value parameters of the problem, the real-valued solution of problem (11)–(13) was obtained. Repeating this procedure of random choice many times, finally, the results were obtained as frequency distributions of optimal real-valued $x_{ij}$ and Pr.

In order to make comparable the results obtained using the fuzzy and Monte–Carlo approaches, the special, simple method for the conversion of frequency distributions into fuzzy values with inevitable but acceptable loss of information was used. This method allows us to guaranty the comparability of uncertain data in the fuzzy and probabilistic cases. To make our analysis more transparent, we used the simple, normally distributed frequency functions, completely represented be their means *m* and standard deviations σ.

The proposed method is implemented in two steps:

In the first one, with the use of initial normally distributed frequency function *f*(*x*), the cumulative distribution function *F*(*x*) is calculated as follows: $F\left(x\right)=\int_{-\infty }^{x}f(x)dx$.

In the second step, a trapezoidal fuzzy number is obtained on the basis of F(x). The four values $F(x_{i})$, *i* = 1 to 4, defining the mapping of F(x) on *X* should be chosen in such a way that they specify the bottom and upper α-cuts of the trapezoidal fuzzy number.

So, we obtain the quadruple on *X*: $\left[x_{1},x_{2},x_{3},x_{4}\right]$ such that interval $\left[x_{1},x_{4}\right]$ represents the bottom of trapeze and interval $\left[x_{2},x_{3}\right]$ is the top of trapeze. Because the probability that *x* is included in the interval $\left[x_{1},x_{4}\right]$ may be calculated as $F\left(x_{4}\right)-F\left(x_{1}\right)$ and the probability that *x* is included in the interval $\left[x_{2},x_{3}\right]$ is equal to $F\left(x_{3}\right)-F\left(x_{2}\right)$, in our example the intervals $\left[x_{2},x_{3}\right]$ and $\left[x_{1},x_{4}\right]$ were chosen in such a way that they corresponded to the 20% (for the top of trapeze) and 80% (for the bottom of trapeze) values of the confidence interval, respectively. Obviously, we placed these intervals in such a way that they were centered around the center of the cumulative distribution F(x).

It is clear that the accuracy of the proposed method of transformation was based only on the subjective opinions of experts concerned with the suitability of upper and lower confidence intervals. Obviously, this subjectivity provides some uncertainty. Nevertheless, we expected that the choice of 20% and 80% confidence intervals would guaranty at least satisfactory results of transformation.

Consider the example.

**Example** **1.** *Let us consider the fully fuzzy distribution problem (11)–(13) in the case of N = 3 and M = 3. To collate the fuzzy solution with that obtained by Monte–Carlo method, all the uncertain parameters were first represented by normal frequency distributions. As it was mentioned above, the parameters $a_{i}$ and $b_{j}$ are real values and in our example they are presented as follows:*

*$a_{1}$ = 500, $a_{2}$ = 490, $a_{3}$ = 590, $b_{1}$ = 405, $b_{2}$ = 485, $b_{3}$ = 585.*

*The uncertain parameters were represented by normal frequency distributions with the means presented in Table 4.*

*For the sake of simplicity, all the standard deviations σ were equal to 12. With the use of proposed method for the transformation of frequency distribution function into a fuzzy value, the trapezoidal fuzzy parameters of problem (11)–(13) were obtained. They are presented in Table 4.*

*The results obtained from the model (11)–(13) using above parameters are presented in Table 5.*

**Figure 3:**
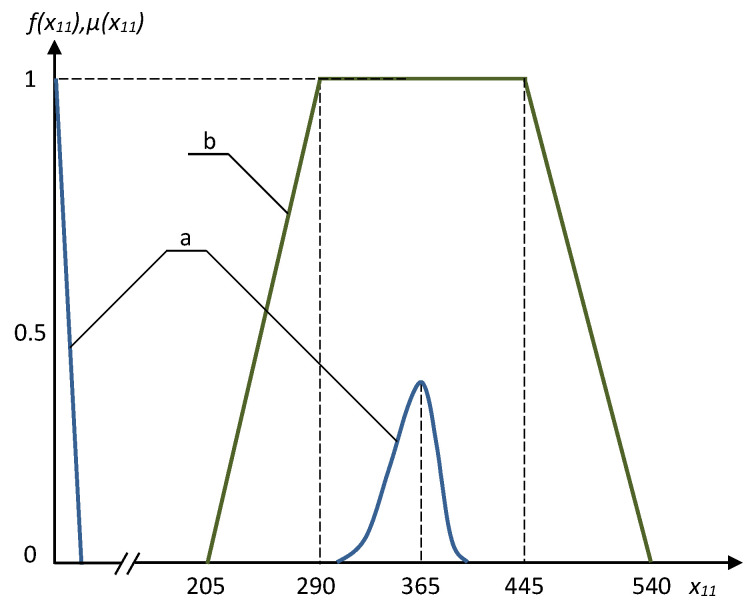
The frequency distribution (**a**) and the membership function of fuzzy value (**b**) for the optimal $x_{11}$.

**Figure 4:**
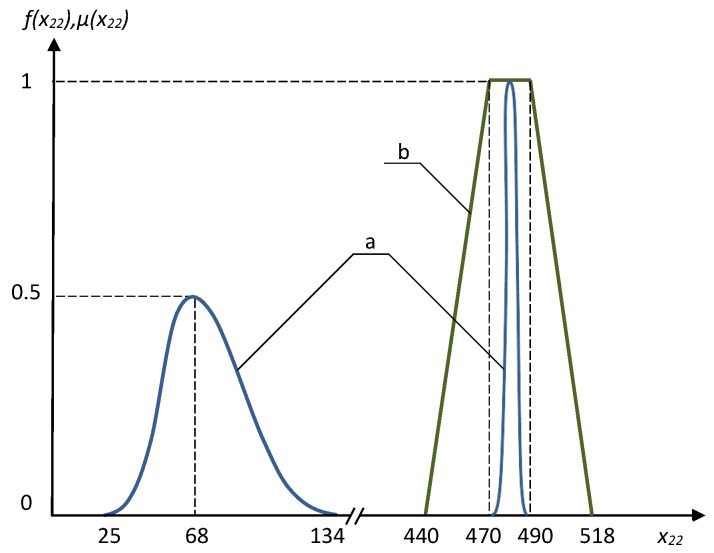
The frequency distribution (**a**) and the membership function of fuzzy value (**b**) for the optimal $x_{22}$.

**Figure 5:**
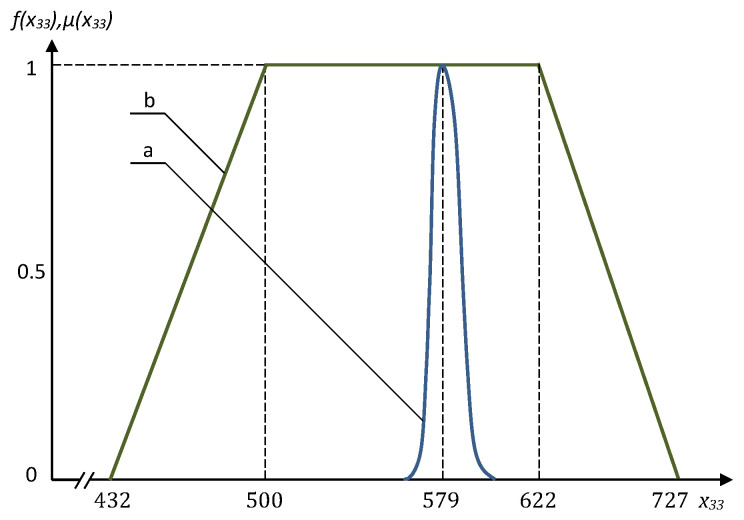
The frequency distribution (**a**) and the membership function of fuzzy value (**b**) for the optimal $x_{33}$.

**Figure 6:**
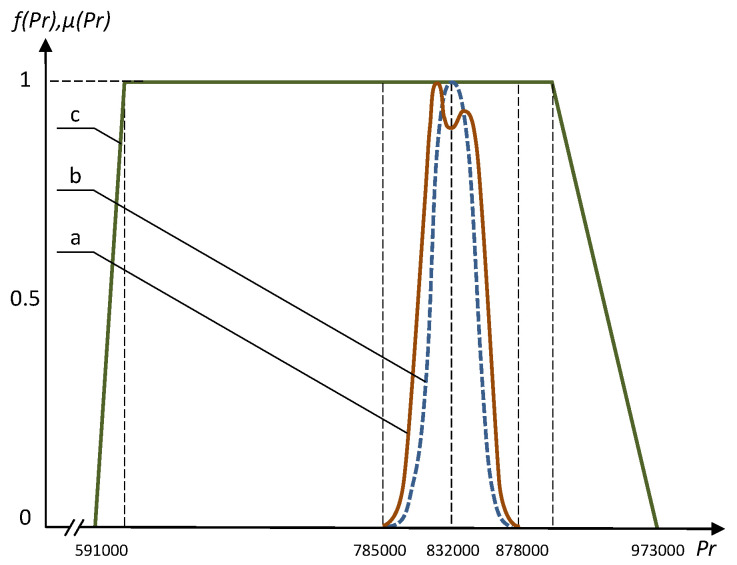
The frequency distribution *f* and the fuzzy number μ for optimized profit $\hat{P}r$: (**a**) Monte–Carlo method on the basis of 10,000 random steps. (**b**) Monte–Carlo method on the basis of one million random steps. (**c**) Membership function of the fuzzy solution.

Several interesting results obtained using the fuzzy approach and Monte–Carlo method are presented in Figure 3, Figure 4, Figure 5 and Figure 6. The frequency distributions in Figure 3, Figure 4 and Figure 5 were obtained with the use of Monte–Carlo method on the basis of one million random steps.

We can see that the Monte–Carlo method sometimes may provide resulting frequency distribution functions with two peaks, and the bigger peaks are in a good qualitative compliance with the results of fuzzy solution (see Figure 3 and Figure 4). The negative effect of multiple peaks in the results of Monte–Carlo method may be eliminated using a much bigger number of random steps (see Figure 6), but this is a very expensive approach. Of course, using the fuzzy optimization, we have no the problem of multiple peaks, as the results are always trapezoidal fuzzy numbers. The resulting fuzzy solutions sometimes are substantially wider than those obtained using Monte–Carlo method (Figure 5). This is the consequence of well known “access width effect” (the results of fuzzy and interval computations are usually wider than the widths of initial data) [17]. On the other hand, when deal with the fuzzy problem, we take into account the values that have extremely low probability in the Monte–Carlo method.

Analyzing the results, we can say that the influence of “access width effect” is not so important. Therefore, the proposed straightforward fuzzy extension of the simplex method may be used for the solution of fully fuzzy distribution problems.

Since the “access width effect” is one of the most important problems of interval and fuzzy arithmetic, the sensitivity analysis that investigates the influence of uncertainty of model’s parameters on the uncertainty of results should to be relevant. To provide such a sensitivity analysis, at least two substantially different examples of the solution of the problem (11)–(13) are needed. Therefore we used the Example 1 and the addition one (Example 2) in the case of four sellers and six buyers.

**Example** **2.** *Let us consider the solution of the problem (11)–(13) in the case of four sellers (M = 4) and six buyers (N = 6). The real-valued parameters in our example are presented as follows:*

*$a_{1}$ = 270, $a_{2}$ = 295, $a_{3}$ = 390, $a_{4}$ = 400, $b_{1}$ = 330, $b_{2}$ = 250, $b_{3}$ = 260, $b_{4}$ = 260, $b_{5}$ = 150, $b_{6}$ = 160.*

*The uncertain parameters are presented in Table 6 and Table 7.*

The solution of this problem obtained using the developed method is presented in Table 8 (only non-zero values are shown).

To study the influence of uncertainty of the model’s fuzzy parameters on the uncertainty of solution, we introduced two uncertainty indexes *UIP* and *UIS*, which represent the averaged uncertainty of model’s parameters and averaged uncertainty of fuzzy solution, respectively. They are defined as follows:

- $UIP=\frac{1}{Np}\sum_{i=1}^{Np}\frac{2(S_{i}^{u}-S_{i}^{l})}{S_{i}^{u}+S_{i}^{l}}$, where Np is the number of uncertain parameters of the model; $S_{i}^{l}$ and $S_{i}^{u}$ are the lower and upper bounds of the support of the *i*’th uncertain parameter.
- $UIS=\frac{1}{Ns}\sum_{j=1}^{Ns}\frac{2(S_{j}^{u}-S_{j}^{l})}{S_{j}^{u}+S_{j}^{l}}$, where Ns is the number of uncertain components of solution; $S_{j}^{l}$ and $S_{j}^{u}$ are the lower and upper bounds of the support of the *j*’th uncertain component of solution.

Of course, different, more complex definitions of *UIP* and *UIS* may be proposed; e.g., based on the averaging on α-cuts. Nevertheless, we prefer to use our simple definitions as they are based on the supports of fuzzy parameters and variables emphasizing our intention to deal with the maximal uncertainty.

Let us turn to our examples.

For Example 1, based on the Table 4 and Table 5, we obtained: *UIP* = 0.1 and *UIS* = 0.4.

For Example 2, based on the Table 6, Table 7 and Table 8, we obtained: *UIP* = 0.3 and *UIS* = 0.5.

Based on the results and comparing dimensionalities of the tasks in the examples considered, we can conclude that uncertainty of the solution rises along with the increasing of uncertainty and the number of parameters. Of course, this is not a surprising conclusion, but more important is that the rising of solution’s uncertainty in the considered examples is relatively small.

Therefore, we can say that in the framework of our approach to the solution of fully fuzzy distribution problem, the influences of the "access width effect " do not prevent using our approach in applications.

## 3. The Formulation of the Multiobjective Fuzzy Distribution Problem

In the first phase of solution of fully fuzzy distribution problem we considered it as a single criterion problem. The overall fuzzy profit with fuzzy constraints was maximized. On the other hand, these constraints may serve as local criteria. This corresponds to general approach to the solution of fuzzy optimization problems developed by Bellman and Zadeh [26].

### 3.1. The Formulation of Problem

Consider the fuzzy parameters ${\hat{p}}_{i}$ and ${\hat{q}}_{i}$, in the fuzzy constraints (13). They are the lowest fuzzy limits for the possible optimal amounts of product to be bought and sold, respectively. Hence, the real-valued $p_{i}$ and $q_{i}$ are established as a result of negotiations. It is important that an appropriate selection of the real values $p_{i}\in {\hat{p}}_{i}$, $q_{i}\in {\hat{q}}_{i}$ strongly affects the overall profit. So we can say that the fuzzy values ${\hat{p}}_{i}$ and ${\hat{q}}_{i}$ may be naturally treated as local criteria.

Let us consider the ${\hat{p}}_{i}$ (the fuzzy bounds for amount of product which can be obtained from the seller). For the sake of simplicity, suppose that ${\hat{p}}_{i}$ is a trapezoidal fuzzy number ${\hat{p}}_{i}$= $\left[p_{i1},p_{i2},p_{i3},p_{i4}\right]$.

Then the interval $\left[p_{i1},p_{i4}\right]$ can be interpreted as the fuzzy interval of acceptable values of $p_{i}$ with the corresponding membership function $\mu_{i}\left(p_{i}\right)$.

Consider the interval $\left[p_{i1},p_{i2}\right]$. In this case, the lessening of $\mu_{i}\left(p_{i}\right)$ along with the decreasing of $p_{i}$ is clearly understood as follows: the risk that contract signed between buyer and distributor will be unfulfilled is rising with the decreasing of $p_{i}$. On the other hand, the rising of $p_{i}$ in the interval $\left[p_{i3},p_{i4}\right]$ leads to the lowering of $\mu_{i}\left(p_{i}\right)$ and as a consequence, to the rising of the overbuying risk (the risk that the part of the bought product could not be sold to the buyer). Obviously, in the interval $\left[p_{i2},p_{i3}\right]$ we have no any risks, since $\mu_{i}\left(p_{i}\right)$ =1. It is worth noting that in such reasoning, the membership function $\mu_{i}\left(p_{i}\right)$ is the representation of the risk varying in the interval [0, 1] and calculated as 1 – $\mu_{i}\left(p_{i}\right)$.

Similarly, the membership function $\mu_{j}\left(q_{j}\right)$ can be interpreted as the corresponding risk belonging to the interval [0, 1] and calculated as 1 – $\mu_{j}\left(q_{j}\right)$.

Therefore, the fuzzy distribution problem can be interpreted as the multiobjective optimization task comprising the local criteria of overall profit maximization and the local risks’ minimization. In our case, the local criteria of a particular risks’ minimization can be formulated straightforwardly using the membership functions of fuzzy values ${\hat{p}}_{i}$ and ${\hat{q}}_{i}$. Nevertheless, an explicit mathematical formalization of the criterion of overall profit maximization can be provided using an additional analysis. In order to formulate this criterion, we used the solution obtained in the first phase in Section 2 with the use of straightforward, direct fuzzy extension of simplex method for the solution of fully fuzzy distribution problem. There are no any additional restrictions on the form of fuzzy parameters and variables in this approach and it is based on the overall profit maximization with fuzzy constraints. Therefore, the optimal fuzzy profit $\hat{P}r$ can be interpreted as the fuzzy interval of all the attainable real-valued profits.

Therefore, to formulate the local criterion *Cpr(Pr)* representing the distributor’s intention to maximize the overall profit, it is enough to use the support of $\hat{P}r$. In the case of trapezoidal fuzzy solution as in the previous section, we can present it by the quadruple $[Pr_{1},Pr_{2},Pr_{3},Pr_{4}].$ Then, the local criterion of overall profit maximization may be presented as follows:

(14) $$Cpr=\frac{Pr-Pr_{1}}{Pr_{4}-Pr_{1}}.$$

It is seen that this criterion does not present the “possibility” to obtain the profit that is implicitly presented by the fuzzy profit $\hat{P}r$ obtained at the first phase using the constraints defined by the fuzzy parameters ${\hat{p}}_{i}$ and ${\hat{q}}_{i}$, because in the multiobjective distribution problem we consider these parameters as local criteria of risk minimization. We can see that for the calculation of *Cpr(Pr)*, we need the real-valued *Pr*.

Obviously, in practice all the bought and sold product quantities $p_{i}$, $q_{j}$ and optimal product quantities $x_{ij}$ supplied from *i*th seller to *j*th buyer according to the signed contracts should be real values. Therefore, some simplifications of the initial fuzzy problem seem to be enough justified. Therefore, we use in (11), the real-valued representations $pr_{ij}$ instead of fuzzy $\hat{p}r_{ij}$. In real-world situations, usually, the prices $c_{i}$, $c_{j}$ and transportation costs of supplying of product unit $t_{ij}$ are well-known real values. Hence, $pr_{ij}$ = $c_{j}$ − $c_{i}$ − $t_{ij}$ are real values too. Therefore, when we deal with the symmetrical trapezoidal fuzzy $\hat{p}r_{ij}$, the means of such trapezes will be used.

These simplifications allow us to get from (11)–(13) for the real-valued $p_{i}\in {\hat{p}}_{i}$, $q_{j}\in {\hat{q}}_{j}$, *i* = 1 to *N*, *j* = 1 to *M*, the real-valued optimal product quantities ${x_{ij}}_{opt}$, and the real-valued profit $Pr=\sum_{i=1}^{M}\sum_{j=1}^{N}\left(pr_{ij}x_{ij_{opt}}\right)$.

It is clear that ${x_{ij}}_{opt}$ depends only on $p_{i}\in {\hat{p}}_{i}$, $q_{j}\in {\hat{q}}_{j}$ and Pr depends on $p_{i}$,$q_{j}$ as well: $Pr(\left\{p_{i}\right\},\left\{q_{j}\right\})$. The values of $Pr(\left\{p_{i}\right\},\left\{q_{j}\right\})$ are needed to calculate the values of local criterion of profit maximization $Cpr(Pr\left(\left\{p_{i}\right\},\left\{q_{j}\right\}\right))$ at the following steps of the proposed algorithm for the solution of fuzzy multiobjective distribution problem.

As it was shown above, the local risks minimization can be formulated as the maximization of membership functions $\mu_{i}\left(p_{i}\right)$, and $\mu_{j}\left(q_{j}\right)$, Therefore, the solution of multiobjective distribution problem are the optimal ${\left\{p_{i}\right\}}_{opt}\in \left\{{\hat{p}}_{i}\right\}$,${\left\{q_{j}\right\}}_{opt}\in \left\{{\hat{q}}_{j}\right\}$ maximizing some generalized criterion aggregating all the considered local criteria $Cpr(Pr\left(\left\{p_{i}\right\},\left\{q_{j}\right\}\right))$, $\mu_{i}\left(p_{i}\right)$, $\mu_{j}\left(q_{j}\right)$ with their relative importance. Then, the optimal product quantities $x_{ij}$ supplied from *i*th seller to *j*th buyer are finally obtained as the solution of problem (11)–(13) for the real-valued $pr_{ij}$ and optimal ${\left\{p_{i}\right\}}_{opt}\in \left\{{\hat{p}}_{i}\right\}$, ${\left\{q_{j}\right\}}_{opt}\in \left\{{\hat{q}}_{j}\right\}$, *i* = 1 to *N*, *j* = 1 to *M*.

### 3.2. The Solution of Multiobjective Fuzzy Distribution Problem Using the Aggregation of Different Aggregating Modes

In the formulation of generalized criterion, we aggregate the criteria of local risks to obtain the aggregated risk minimization criterion and aggregate this risk criterion with the profit maximization one. Among many approaches to the formulation of the general criterion presented in the literature, the most popular are the multiplicative aggregation mode, Yager’s [27] aggregation, and the weighted sum (see [28]). It is known that the choice of the relevant aggregating method is a context dependent problem [14]. In our case, the most popular aggregating modes may be presented as follows:

(15) $$\begin{array}{c} E_{1}\left(\left\{p_{i}\right\},\left\{q_{j}\right\}\right)=\alpha \cdot Cpr\left(Pr\left(\left\{p_{i}\right\},\left\{q_{j}\right\}\right)\right)+\left(1-\alpha \right)\cdot (\mu_{1}\left(p_{1}\right)+\mu_{2}\left(p_{2}\right)+\ldots +\\ \mu_{M}\left(p_{M}\right)+\mu_{1}\left(q_{1}\right)+\mu_{2}\left(q_{2}\right)+\ldots +\mu_{N}\left(q_{N}\right))/\left(2*\left(N+M\right)\right),\\ E_{2}\left(\left\{p_{i}\right\},\left\{q_{j}\right\}\right)=\min(Cpr^{\alpha }\left(Pr\left(\left\{p_{i}\right\},\left\{q_{j}\right\}\right)\right),\min(\mu_{1}\left(p_{1}\right),\mu_{2}\left(p_{2}\right),\ldots ,\\ \mu_{M}\left(p_{M}\right),\mu_{1}\left(q_{1}\right),\mu_{2}\left(q_{2}\right),\ldots ,\mu_{N}\left(q_{N}\right)){}^{1-\alpha }),\\ E_{3}\left(\left\{p_{i}\right\},\left\{q_{j}\right\}\right)=Cpr^{\alpha }\left(Pr\left(\left\{p_{i}\right\},\left\{q_{j}\right\}\right)\right)\cdot (\mu_{1}\left(p_{1}\right)\cdot \mu_{2}\left(p_{2}\right)\cdot \ldots \\ \cdot \mu_{M}\left(p_{M}\right)\cdot \mu_{1}\left(q_{1}\right)\cdot \mu_{2}\left(q_{2}\right)\cdot \ldots \cdot \mu_{N}\left(q_{N}\right)){}^{1-\alpha }), \end{array}$$

where 0≤α≤1 is the relative importance of the profit maximization criterion, $E_{1}$ is the weighted sum aggregation, $E_{2}$ is the Yager’s aggregation, and $E_{3}$ is the multiplicative aggregation. The relative importance of each risks’ local minimization criteria in our case were equal, as we had no reasons for another assumption. Therefore, we used the aggregation of risk criteria with the common relative importance 1−α. Then, the optimal alternatives ${\left(\left\{p_{i}\right\},\left\{q_{j}\right\}\right)}_{k,opt}$, *k* = 1, 2, 3 for the general criteria $E_{1}$, $E_{2}$, $E_{3}$ may be obtained as the solutions of the following nonlinear multiobjective optimization problems:

(16) $$\max\left(E_{k}\left(\left\{p_{i}\right\},\left\{q_{j}\right\}\right)\right),k=1,2,3,\left\{p_{i}\right\}\in \left\{{\hat{p}}_{i}\right\},\left\{q_{j}\right\}\in \left\{{\hat{q}}_{j}\right\}.$$

Based on the formal mathematical approach with the use of corresponding theorem and illustrative examples, it was shown in [28] that the most correct aggregation mode is the Yager’s type aggregation ($E_{2}$); the multiplicative aggregation ($E_{3}$) seems to be less reliable; and finally, the weighted sum ($E_{1}$) may be treated as rather unreliable one. Nevertheless, it is known that Yager’s aggregation sometimes provides results which contradict with intuitive conceptions of experts concerned with the optimality [29].

Thus, when we a difficult multiobjective problem with a great number of local criteria, applying all feasible aggregating modes is sufficiently justifiable. If the results of optimization obtained using different aggregation modes are comparable, we can believe that they are rather optimal ones. In the case, when the results we get with the use of different aggregating modes are substantially different ones, the compromise solution based on the appropriate aggregation of aggregating modes may be recommended.

The different methods for aggregation of aggregating modes were proposed in the literature References [30,31,32]. The weighted sum, Yager’s aggregation, multiplicative aggregation, and the different combinations of them are used in aggregation of aggregating modes. The most important drawback of these methods is that they do not provide the aggregation of all feasible aggregating modes.

In [28], a simple, but intuitively evident and mathematically justified approach which makes it possible to avoid the above-mentioned drawback was developed. The proposed method provides the aggregation of aggregating modes with the use of mathematical tools of the level-2 fuzzy sets. Therefore, in the current study, we used this method.

The definition of the level-2 fuzzy sets was formulated by Zadeh in [33] as follows: “The level-2 fuzzy set is such a fuzzy set, which membership grades assigned to the elements of the universal set are ordinary fuzzy sets.” The method developed in [28] was designed to solve decision making problems. Therefore, here, we will extend it for the solution of the multiobjective optimization problems.

Obviously, the solution of our nonlinear multiobjective optimization task can be provided only by the use of numerical methods. Therefore, when implementing such a method we will use the discrete finite set of alternatives $al_{k}={\left(\left\{p_{i}\right\},\left\{q_{j}\right\}\right)}_{k}$, *k* = 1 to *L*, where *L* is the number of step of an algorithm (it is clear that in the optimization tasks we usually deal with the continuous sets of alternatives, but here we will use the discrete finite set to make our consideration more transparent). Such an algorithm provides the searching for the optimal alternative $al_{Lopt}={\left(\left\{p_{i}\right\},\left\{q_{j}\right\}\right)}_{Lopt}$ in the area $p_{i}\in {\hat{p}}_{i}$, $q_{j}\in {\hat{q}}_{j}$, *i* = 1 to *N*, *j* = 1 to *M*.

Then, suppose we deal with *K* different aggregating modes $F_{l}$, *l* = 1 to *K*, and *L* alternatives $al_{k}={\left(\left\{p_{i}\right\},\left\{q_{j}\right\}\right)}_{k}$, *k* = 1 to *L*. Let us assume that the membership functions $\mu (E_{l})$, *l* = 1 to *K*, reflect the opinion of expert concerning proximity of considering aggregation mode $E_{l}$ to the some perfect (“ideal”) type of aggregation. The values of such membership functions may be assumed to be the relative reliabilities of aggregating modes. On the other hand, the “ideal” method of aggregation $E_{ideal}$ can be represented by the fuzzy set using the membership functions $\mu (E_{l})$ and the set of considered aggregating modes $E_{l}$ as follows:

(17) $$E_{ideal}=\left\{\frac{\mu (E_{l})}{E_{l}}\right\},\;l=1\;to\;K.$$

Then, all $E_{l}$ can be formally defined on the set of considered alternatives $al_{k}={\left(\left\{p_{i}\right\},\left\{q_{j}\right\}\right)}_{k}$, *k* = 1 to *L*, for which the values of $E_{l}\left(al_{k}\right)$ are factually calculated. In turn, the value of $E_{l}\left(al_{k}\right)$ may be naturally interpreted as an extent in which the alternative $al_{k}$ fulfills the aggregated criterion $E_{l}$ or as a degree in which the alternative $al_{k}$ pertains to a set of alternatives fulfilling $E_{l}$.

(18) $$E_{l}=\left\{\frac{E_{l}\left(al_{k}\right)}{al_{k}}\right\},\;k=1\;to\;L.$$

Then, substituting (18) in (17) we get the expression for the $E_{ideal}$ in the form of level-2 fuzzy set; and with the use of operations defined on these fuzzy sets in [33], we finally obtain:

(19) $$E_{ideal}=\left\{\frac{\mu_{ideal}\left(al_{k}\right)}{al_{k}}\right\},\;k=1\;to\;L,$$

where

(20) $$\mu_{ideal}\left(al_{k}\right)=\max_{l}\left(\mu \left(E_{l}\right)\cdot E_{l}\left(al_{k}\right)\right).$$

Finally, the optimal alternative $al_{opt}$ can be obtained as the solution of the following non-linear fuzzy multiobjective problem:

(21) $$\max_{k}\mu_{ideal}\left(al_{k}\right).$$

For the solution of the formulated nonlinear fuzzy multiobjective optimization problem, we applied the direct random search method [34], which was adopted to take into account the features of our task. Obviously, there are many other contemporary optimization methods; e.g., genetic algorithms proposed in the literature. However, using different convincing examples, it is shown in [35] that if we deal with the nonlinear, non-differentiable, or non-smooth optimization problem the direct search methods provide the best results.

The developed algorithm of direct random search method has been implemented as follows:

1. In each *k*th random step, the alternative $al_{k}={\left(\left\{p_{i}\right\},\left\{q_{j}\right\}\right)}_{k}$ is randomly chosen in the area $p_{i}\in {\hat{p}}_{i}$, $q_{j}\in {\hat{q}}_{j}$.
2. For the chosen ${\left(\left\{p_{i}\right\},\left\{q_{j}\right\}\right)}_{k}$ and the real-valued representations of $\hat{p}r_{ij}$ from (11)–(13), the profit $Pr_{k}$ and product quantities ${\left\{x_{ij}\right\}}_{k}$ are computed.
3. This allows us to obtain the value of the local criterion $Cpr(Pr_{k})$ (14) and the values of $E_{l}\left(al_{k}\right)$, *l* = 1 to *K* (15). Here, we consider only the weighted sum ($E_{1}$), Yager’s ($E_{2}$), and multiplicative ($E_{3}$) aggregations.
4. Finally, from (20) we obtain the value of $\mu_{ideal}\left(al_{k}\right)$.
5. If $\mu_{ideal}\left(al_{k}\right)>\mu_{ideal}\left(al_{k-1}\right)$, we consider the *k*th step of algorithm as the successful one. Then, continuing the procedure of random search, we, step by step, steadily approach the optimal $\mu_{ideal}\left(al_{opt}\right)$.

**Figure 7:**
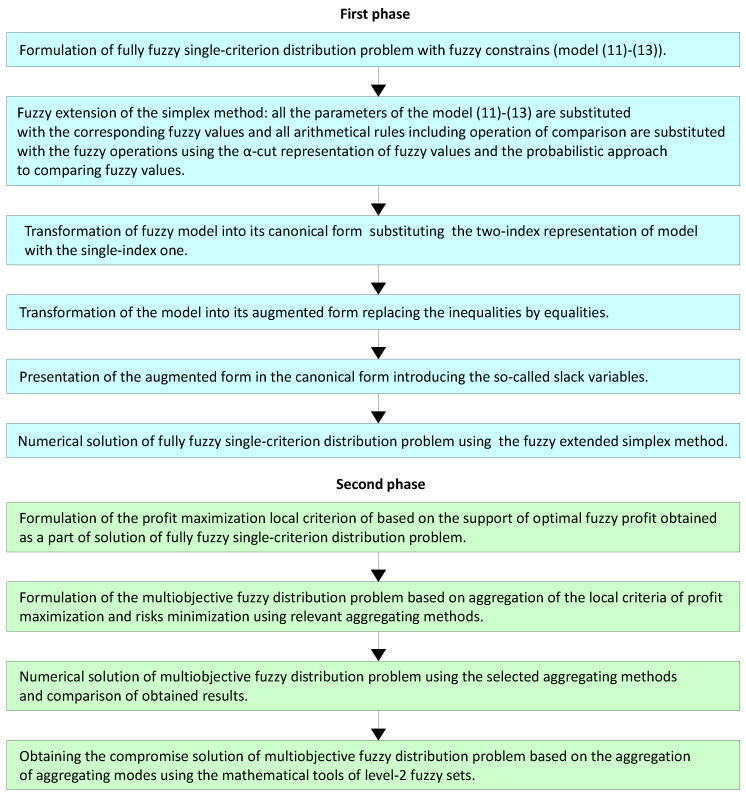
The enlarged flowchart of the developed method.

The enlarged flowchart of the developed two phase method for multiobjective fuzzy distribution problem is presented in Figure 7.

Consider the numerical example.

Here we will use the Example 1 from Section 2 instead of fuzzy $\hat{p}r_{ij}$ and the real-valued representations $pr_{ij}$. Since in our case, we deal with the symmetrical trapezoidal fuzzy profits $\hat{p}r_{ij}$, the means of the trapezes were used.

In Table 9, the compromise solution of multiobjective fuzzy distribution problem based on the aggregation of aggregating modes is compared with the results that we got using the weighted sum ($E_{1}$), Yager’s ($E_{2}$), and multiplicative ($E_{3}$) aggregating modes with the use of the adapted, direct random search method.

Based on the proposition justified in [28], the following reliability degrees of aggregating modes were used: $\mu (E_{1})=0.15$, $\mu (E_{2})=0.6$, $\mu (E_{3})=0.25$.

Finally, comparing the results presented in Table 9, we can conclude that solution obtained using the aggregation of aggregating modes can be considered as a compromise one, because it is located in the domain of solutions we obtained solely by the comparing aggregating modes.

## 4. Conclusions

A two-phase method for the solution of multiojective fuzzy distribution problem was proposed. In the first stage, the straightforward numerical method for the solution of single-criterion fully fuzzy distribution problem (*FFDP*) was developed. The α-cut representation of fuzzy values, fuzzy operation rules, and the probability approach to the intervals and fuzzy values comparison are used as the main mathematical tools. These tools allow us to implement the straightforward fuzzy extension of the ordinary simplex method, which is used for the solution of the single-criterion *FFDP*. To validate the method, the fully fuzzy transportation problem with triangular fuzzy numbers was solved in the case of three sources and four destinations. The results were compared with those obtained by the competing method. As the base of comparison, the results of the single-criterion *FFDP* solution obtained with the use of Monte–Carlo method were also applied. To make the results of Monte–Carlo method and fuzzy approach we developed comparable, the frequency distributions of uncertain input parameters were transformed into trapezoidal fuzzy values, which were used in the solution of the single-criterion *FFDP*. Taking into account that the used data transformation leads inevitable to the loss of some information, the results of comparison of two considered approaches (fuzzy and Monte–Carlo ones) may be recognized as at least satisfactory ones. We can say that the fuzzy approach possesses some advantages when compared with Monte–Carlo method, mainly from the computing costs point of view.

The sensitivity analysis of our optimization model developed for the solution of *FFDP* was also carried out using numerical examples.

Finally, based on the provided studies, we can conclude that the method developed for the solution of *FFDP* has some advantages in comparison with the competing approach and may be used in applications.

In the second phase, the support of optimal fuzzy profit obtained at the first phase is treated as the range of attainable real-valued profits and used in the formulation of particular criterion of the overall profit maximization. The fuzzy constraints are interpreted as particular criteria of risk minimization. The generalized criterion was designed in the form of aggregation of relevant aggregating modes with the use of level-2 fuzzy sets. The aggregating modes represent different methods for aggregations of local criteria defining the overall profit and contract-violating risks. It was shown based the numerical example that the solution we obtained on the base of developed method for the aggregation of aggregating modes can be assumed as the compromise solution because it is located in the domain of solutions we obtained using solely the comparison of aggregation modes.

The main limitation of the approach developed for the solution of *FDP* is that in the first phase we obtain the fuzzy solution, whereas at the second one we get the real-valued solution of fuzzy multiobjective problem. Obviously, in our case the real-valued solution of fuzzy problem may be considered only as an approximate one due to possible loss of important information.

Therefore, our future studies will be focused on the finding of fuzzy solution of *FDP* formulated as a nonlinear fuzzy multiobjective problem.

## Figures and Tables

**Table 1:** Triangular fuzzy values of the model’s parameters.

Parameters	Fuzzy Values	Parameters	Fuzzy Values
${\hat{a}}_{1}$	[7.2, 8, 8.8]	${\hat{c}}_{11}$	[8, 10, 10.8]
${\hat{a}}_{2}$	[12, 14, 16]	${\hat{c}}_{12}$	[20.4, 22, 24]
${\hat{a}}_{3}$	[10.2, 12, 13.8]	${\hat{c}}_{13}$	[8, 10, 10.6]
${\hat{b}}_{1}$	[6.2, 7, 7.8]	${\hat{c}}_{14}$	[18.8, 20, 22]
${\hat{b}}_{2}$	[8.9, 10, 11.1]	${\hat{c}}_{21}$	[14, 15, 16]
${\hat{b}}_{3}$	[6.5, 8, 9.5]	${\hat{c}}_{22}$	[18.2, 20, 22]
${\hat{b}}_{4}$	[7.8, 9, 10.2]	${\hat{c}}_{23}$	[10, 12, 13]
		${\hat{c}}_{24}$	[6, 8, 8.8]
		${\hat{c}}_{31}$	[18.4, 20, 21]
		${\hat{c}}_{32}$	[9.6, 12, 13]
		${\hat{c}}_{33}$	[7.8, 10, 10.8]
		${\hat{c}}_{34}$	[14, 15, 16]

**Table 2:** Fuzzy efficient solutions on α-cuts.

$\left[x_{\mathrm{ij}}\right]$	α=0	α=0.5	α=1
$\left[x_{11}\right]$	[6.2, 7.8]	[6.6, 7.4]	[7, 7]
$\left[x_{13}\right]$	[1, 1]	[1, 1]	[1, 1]
$\left[x_{23}\right]$	[4.2, 5.8]	[4.6, 5.4]	[5, 5]
$\left[x_{24}\right]$	[7.8, 10.2]	[8.4, 9.6]	[9, 9]
$\left[x_{32}\right]$	[8.9, 11.1]	[9.5, 10.6]	[10, 10]
$\left[x_{33}\right]$	[1.3, 2.7]	[1.7, 2.4]	[2, 2]

**Table 3:** Fuzzy efficient solutions on α-cuts (our method).

$\left[x_{\mathrm{ij}}\right]$	α=0	α=0.5	α=1
$\left[x_{11}\right]$	[6.4, 8.0]	[6.5, 7.1]	[7, 7]
$\left[x_{13}\right]$	[1, 1]	[1, 1]	[1, 1]
$\left[x_{23}\right]$	[4.0, 5.6]	[4.4, 5.6]	[5, 5]
$\left[x_{24}\right]$	[7.4, 10.3]	[8.2, 9.8]	[9, 9]
$\left[x_{32}\right]$	[8.6, 10, 9]	[9.6, 10.9]	[10, 10]
$\left[x_{33}\right]$	[1.1, 2.9]	[1.6, 2.6]	[2, 2]

**Table 4:** The values of model’s parameters.

The Means		The Fuzzy Values	
$p_{1}$	425	${\hat{p}}_{1}$	[402, 420, 439, 444]
$p_{2}$	452	${\hat{p}}_{2}$	[429, 447, 456, 471]
$p_{3}$	600	${\hat{p}}_{3}$	[590, 598, 607, 622]
$q_{1}$	402	${\hat{q}}_{1}$	[379, 397, 406, 421]
$q_{2}$	477	${\hat{q}}_{2}$	[454, 472, 481, 496]
$q_{3}$	602	${\hat{q}}_{3}$	[579, 597, 606, 613]
$pr_{11}$	290	$\hat{p}r_{11}$	[267, 285, 294, 309]
$pr_{12}$	510	$\hat{p}r_{12}$	[487, 505, 514, 529]
$pr_{13}$	505	$\hat{p}r_{13}$	[482, 500, 507, 524]
$pr_{21}$	395	$\hat{p}r_{21}$	[372, 390, 299, 314]
$pr_{22}$	565	$\hat{p}r_{22}$	[546, 564, 573, 585]
$pr_{23}$	283	$\hat{p}r_{23}$	[257, 273, 282, 297]
$pr_{31}$	295	$\hat{p}r_{31}$	[272, 290, 299, 314]
$pr_{32}$	401	$\hat{p}r_{32}$	[380, 398, 407, 422]
$pr_{33}$	615	$\hat{p}r_{33}$	[591, 609, 618, 633]

**Table 5:** The result of solution of fully fuzzy distribution problem (11)–(13).

Solution’s Components	Fuzzy Values
$\hat{P}r_{opt}$	[591,000, 612,000, 900,000, 973,000]
${\hat{x}}_{11}$	[205, 290, 445, 540]
${\hat{x}}_{12}$	[37, 42, 53, 62]
${\hat{x}}_{13}$	[5, 6, 8, 9]
${\hat{x}}_{21}$	0
${\hat{x}}_{22}$	[440, 470, 490, 518]
${\hat{x}}_{23}$	[0, 1, 1, 2]
${\hat{x}}_{31}$	0
${\hat{x}}_{32}$	0
${\hat{x}}_{33}$	[432, 500, 662, 727]

**Table 6:** The fuzzy values of model’s parameters.

i	${\hat{p}}_{i}$	${\hat{q}}_{i}$
**1**	[290, 295, 305, 320]	[320, 340, 360, 365]
**2**	[200, 220, 270, 290]	[200, 240, 260, 280]
**3**	[380, 390, 405, 430]	[200, 240, 260, 280]
**4**	[420, 440, 460, 470]	[200, 240, 260, 280]
**5**		[120, 140, 160, 160]
**6**		[120, 140, 160, 160]

**Table 7:** The fuzzy values of model’s parameters.

	$\hat{p}r_{\mathrm{ij}}$					
i/j	1	2	3	4	5	6
1	[40, 90, 110, 120]	[250, 270, 290, 320]	[330, 330, 560, 370]	[200, 205, 215, 220]	[110, 120, 140, 160]	[260, 280, 300, 310]
2	[60, 65, 75, 90]	[130, 150, 150, 170]	[200, 205, 215, 240]	[100, 120, 135, 150]	[30, 35, 45, 100]	[200, 200, 230, 270]
3	[200, 220, 250, 260]	[320, 340, 355, 360]	[450, 460, 490, 490]	[360, 370, 390, 395]	[250, 260, 280, 330]	[400, 405, 415, 420]
4	[30, 40, 60, 70]	[10, 10, 10, 50]	[220, 225, 240, 250]	[120, 130, 150, 150]	[70, 70, 90, 100]	[250, 260, 280, 290]

**Table 8:** The result of solution of fully fuzzy distribution problem (11)–(13) in the case of *M* = 4, *N* = 6.

Solution’s Components	Fuzzy Values
${\hat{x}}_{12}$	[104, 129, 159, 214]
${\hat{x}}_{13}$	[253, 293, 313, 333]
${\hat{x}}_{21}$	[260, 300, 400, 440]
${\hat{x}}_{32}$	[6, 123, 203, 273]
${\hat{x}}_{34}$	[10, 240, 360, 500]
${\hat{x}}_{35}$	[0, 6, 118, 298]
${\hat{x}}_{41}$	[2, 20, 100, 140]
${\hat{x}}_{45}$	[110, 130, 170, 190]
${\hat{x}}_{46}$	[101, 176, 226, 291]

**Table 9:** The results of solution.

	${\mathrm{Pr}}_{\mathrm{opt}}$	$p_{1}$	$p_{2}$	$p_{3}$	$q_{1}$	$q_{2}$	$q_{3}$	$x_{11}$	$x_{12}$	$x_{13}$	$x_{21}$	$x_{22}$	$x_{23}$	$x_{31}$	$x_{32}$	$x_{33}$
$\mu_{ideal}\left(al_{opt}\right)$	745868	427	458	613	400	487	597	365	49	7	0	485	1	0	0	662
$E_{1}\left(al_{opt}\right)$	749710	440	450	600	380	473	603	308	48	0	0	499	0	0	0	687
$E_{2}\left(al_{opt}\right)$	743809	421	469	620	410	502	608	408	51	1	0	468	3	2	0	679
$E_{3}\left(al_{opt}\right)$	744460	402	430	618	395	480	580	256	50	0	0	489	0	0	0	630
